# PD-L1/PD-1 blockage enhanced the cytotoxicity of natural killer cell on the non-small cell lung cancer (NSCLC) by granzyme B secretion

**DOI:** 10.1007/s12094-023-03120-w

**Published:** 2023-03-01

**Authors:** Duan-Rui Qiao, Jun-Ya Cheng, Wei-Qun Yan, Hai-Jun Li

**Affiliations:** 1 Department of Bioengineering, Pharmacy School of Jilin University, Changchun, 130021 Jilin China; 2 Institute of Translational Medicine, The First Hospital of Jilin University, Changchun, 130021 Jilin China; 3 Institute of Liver Diseases, The First Hospital of Jilin University, Changchun, Jilin China

**Keywords:** PD-L1, PD-1, NK cells, Non-small cell lung cancer, Granzyme B

## Abstract

**Objective:**

To explore the role of PD-L1/PD-1 blockage in the cytotoxicity of natural killer cell in NSCLC.

**Methods:**

Two NSCLC cell lines, Calu-1 and H460, were tested for susceptibility to the cytolytic activity of freshly isolated healthy donor NK cells by a non-radioactive cellular cytotoxicity assay kit. Western blot analysis, FACS, ELISA and antibody blockage experiments were conducted to determine the mechanisms. NK cells isolated from NSCLC patients were also collected for functional assays.

**Results:**

Calu-1 and H460 cells were lysed by NK cells in a dose-dependent manner. H460 cells showed less susceptibility to NK cell-mediated lysis than Calu-1 cells at all ratios. The expression of PD-L1 on H460 cells was higher than that on Calu-1 cells, as determined by FACS and western blot analysis. The specific lysis of H460 cells by NK cells was enhanced when the PD-L1/PD-1 interaction was blocked by anti-PD-L1 antibody. This finding was also demonstrated in NK cells isolated from NSCLC patients.

**Conclusions:**

The present study revealed that PD-L1/PD-1 blockage enhanced the cytotoxicity of natural killer cells in NSCLC via granzyme B secretion. This study will greatly facilitate the precise treatment of lung cancer through determination of PD-L1 expression in tumors.

## Introduction

Lung cancer has the highest incidence and is the leading cause of cancer-related death worldwide [1, 2]. In patients with advanced lung disease, the 1-year, 2-year and 5-year survival rates were 35, 20 and 17.8%, respectively, in a recent study [3]. More concerningly, only half of patients with advanced-stage disease have a 3.7% chance of surviving for 5 years [4]. Non-small cell lung cancer (NSCLC) accounts for approximately 80% of all lung cancer cases. Palliative therapeutic options are used in most NSCLC patients, because most of these cancers are metastatic and at stage III-IV when diagnosed [5]. Treatment of patients with surgery and chemotherapy prolongs survival only modestly but is associated with a wide range of undesirable side effects [6, 7]. Therefore, efforts to develop more effective therapeutic strategies for advanced NSCLC are an area of active investigation.

For patients who are not candidates for surgery, immunotherapy is a promising therapeutic option [8, 9]. Expansion of autologous tumor-specific effector cells ex vivo before infusion into a host plays an important role in adoptive cell immunotherapy [10]. CIK cells, lymphokine-activated killer cells (LAKs), tumor-infiltrating lymphocytes (TILs), cytotoxic T lymphocytes (CTLs) and natural killer (NK) cells are the candidate immunological effector cells for treating cancer alone or in combination use after surgery [11, 12]. There have been promising and encouraging clinical results in the treatment of breast [13], liver [14], and digestive tract [15] cancers with adoptive NK cells. Therefore, the possibility of treating NSCLC with NK cells has attracted increasing attention in the field of cancer immunotherapy. Unlike T cells, NK cells are innate immune lymphocytes that kill or lyse malignant cells by producing cytokines and chemokines in a manner independent of antigen presentation [16, 17].

With the development of immune checkpoint inhibitors for the clinical treatment of malignant tumors, cancer immunotherapy has become an increasingly popular treatment modality. Programmed death ligand-1 (PD-L1), a widely studied immune checkpoint molecule, plays an important role in tumor escape from immunosurveillance [18, 19]. Programmed death-1 (PD-1), a receptor for PD-L1, is expressed on immune cells, including NK cells, and interacts with tumor cells, leading to apoptosis, anergy or tolerance. An increasing number of clinical trials for anti-PD-L1 antibody therapy were approved in China after anti-PD-1 antibodies were approved by the FDA for the treatment of melanoma [20, 21]. In addition, studies have reported that the tumor response to PD-L1 or PD-1 inhibition is directly related to the level of PD-L1 expression and lymphocytic infiltration of the tumor [22–24]. Although PD-1 immune checkpoint inhibitors are effective in 10–20% of lung cancer patients, therapeutic effects are not observed in the remaining patients. The present study aimed to investigate the function and mechanism of NK cells in suppressing the tumorigenicity of NSCLC cell lines in vivo. We sought to determine whether the difference in the expression level of PD-L1 on different NSCLC cell lines results in different outcomes. The results may provide a potential therapeutic approach combining an anti-PD-L1 antibody with NK cells in lung cancer with high PD-L1 expression.

## Material and methods

### Cell isolation and purification

Peripheral blood mononuclear cells (PBMCs) were freshly isolated from the peripheral blood of healthy individuals visiting Changchun Blood Center or from NSCLC patients in the First Hospital of Jilin University via Ficoll density gradient separation. The study was approved by the Ethics Committee of Changchun Blood Center (Ethics No. 2020–026, 2020.01–2020.12) and The First Hospital of Jilin University (Ethics No. 2020–084, 2020.06–2021.12). All experiments were conducted in accordance with the approved guidelines and regulations. Ficoll density gradient separation was performed at a speed of 500 g (acceleration set at 9, deceleration set at 1) for 30 min at room temperature. NK cells were enriched through negative selection from outflow cells with an NK cell isolation kit (^#^130–092-657, Miltenyi Biotec, Bergisch Gladbach, Germany). The purity of the purified cells was equal to or greater than 95%, as determined by flow cytometry.

### Tumor cell lines

The Calu-1 and H460 NSCLC cell lines were purchased from ATCC (Shanghai, China). Cells were cultured in a 96-well plate containing 200 µL of complete medium (DMEM supplemented with 10% FCS, penicillin, streptomycin and glutamine) at 1 × 10^5^ cells/well.

### Cellular cytotoxicity assay

NK cell-mediated cytotoxicity was evaluated using a non-radioactive cellular cytotoxicity assay kit. Tumor cells in a round-bottom 96-well plate were challenged with 100 μL of NK cells at effector:target ratios of 0.1, 0.5, 2.0 and 5.0:1 for 40 min at 37 ℃ in 5% CO_2_. The detailed procedure was performed as described previously [25].

### Flow cytometry

Annexin V-FITC and PI double staining followed by flow cytometric analysis was employed to assess cell apoptosis. Calu-1 and H460 cells were collected at the experimental endpoint. Apoptosis was analyzed using a flow cytometer (FACScan; BD Biosciences) and FlowJo 7.6 FACS analysis software (FlowJo LLC, Ashland, OR, USA). A PE-Cy7 mouse anti-human PD-L1 antibody (Clone: MIH-1) was used for surface staining on Calu-1 and H460 cells. Brefeldin A (Sigma‒Aldrich) was subsequently added at a final concentration of 5 µg/mL. NK cells were stained to evaluate surface expression of CD107a and intracellular expression of Granzyme B and IFN-γ. To investigate the involvement of the selected molecules, blocking experiments were performed by adding the following mAbs: anti-PD-L1, anti-IFN-γ and anti-Granzyme B. Control experiments were performed using isotype-matched mouse antibodies (all mAbs were obtained from R&D Systems). All mAbs were used at a final concentration of 10 µg/mL.

Freshly isolated PBMCs from 10 healthy donors and 10 NSCLC patients were stained with the following antibodies for detection of NK cells: FITC mouse anti-human CD3 (^#^561,806), PE-Cy5 mouse anti-human CD56 (^#^561,904), PE mouse anti-human PD-1 (^#^560,795), BV510 mouse anti-human NKp30 (^#^743,170), APC mouse anti-human NKp46 (^#^558,051), and PE-Cy7 mouse anti-human NKG2D (^#^562,365). All antibodies used for FACS were obtained from BD Biosciences. NK cells isolated from 5 NSCLC patients were purified and cultured with Calu-1 and H460 cells for cytotoxicity assessment. NK cells were cultured with H460 cells with or without an anti-PD-L1 antibody for functional assessment.

### Western blotting

Total cellular protein was extracted, and the protein concentration was determined according to the manufacturer’s protocol. A total of 5–40 μg of protein was separated by 10% SDS-PAGE and then electrophoretically transferred to polyvinylidene fluoride membranes (0.45 µm; EMD Millipore, Billerica, MA, USA) and blocked at 37 °C for 1 h with 5% skim milk in Tris-buffered saline (TBS) with Tween 20 (0.1%). Subsequently, membranes were incubated with monoclonal antibodies against PD-L1 (^#^13,684, 1:1000) and β-actin (^#^3700, 1:1000) at 4 °C overnight. Protein expression levels were determined semiquantitatively by densitometric analysis with Quantity One software (V4.62, Bio-Rad Laboratories, Inc., Hercules, CA, USA).

### Enzyme-linked immunosorbent assay (ELISA)

The cell culture supernatant was collected in each experimental condition. Granzyme B and IFN-γ concentrations were measured by ELISA according to the manufacturer’s instructions.

### Statistical analysis

All analyses were performed with data and results from at least three replicate measurements and are presented as the mean ± SD values. Mean values were compared using an unpaired t test (two groups) or by ANOVA with the Bonferroni correction for multiple comparisons. *P* values < 0.05 were considered to indicate significance. All statistical tests were performed with Prism software (V5.0, GraphPad, San Diego, CA, USA).

## Results

### Two NSCLC cell lines showed distinctive susceptibility to NK cell-mediated lysis

**Fig. 1:**
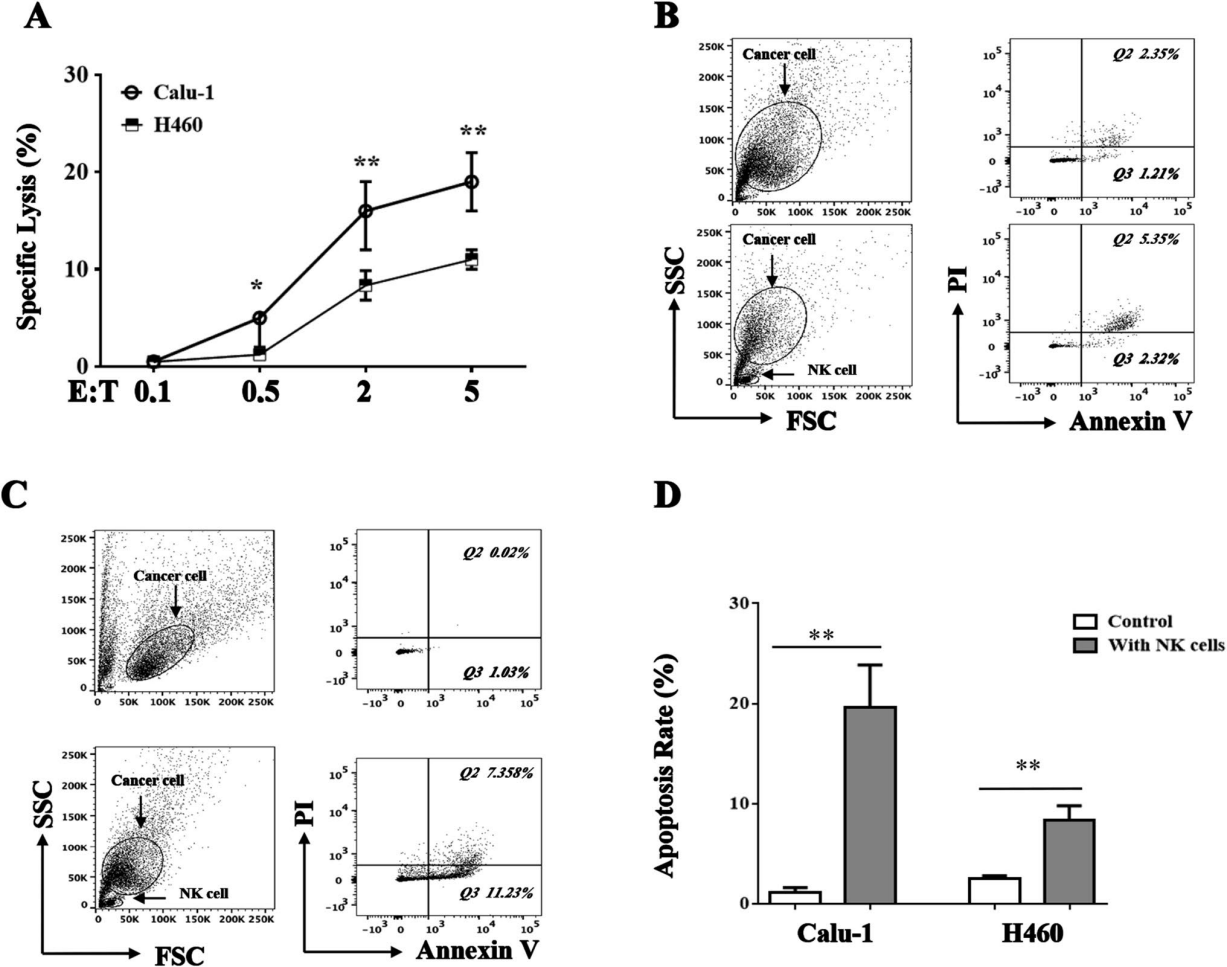
Two NSCLC cell lines showed distinctive susceptibility to NK cells. H460 and Calu-1 cells were plated in the 96-well plate 24 h before NK cells added. Effector-to-target ratios is 0.1, 0.5, 2.0 and 5.0: 1. NK cell-mediated cellular cytotoxicity was determined using a non-radioactive cellular cytotoxicity assay kit **A**. Flow cytometric analysis of apoptosis in H460 and Calu-1 cells treated with NK cell on E:T = 5:1. Tumor cells cultured alone were used as the control. Representative figure of flow cytometric analysis of apoptosis of H460 **B** and Calu-1 **C** cells. Statistical analysis of percentage of apoptotic cells in three independent array **D**. The data are expressed as the mean ± the standard deviation of three experiments. *p < 0.05 and **p < 0.01 vs. control

Two NSCLC cell lines, Calu-1 and H460, were tested for susceptibility to the cytolytic activity of freshly isolated healthy donor NK cells. All cell lines were lysed by NK cells in a dose-dependent manner (effector:target ratios of 0.1, 0.5, 2.0 and 5.0:1). The susceptibility of Calu-1 and H460 cells to the cytolytic activity of NK cells was comparable (*p* > 0.05). Interestingly, H460 cells showed less susceptibility to NK cell-mediated lysis than Calu-1 cells at all ratios (Fig. 1A, *p*  <  0.05). Annexin V-FITC and PI double staining followed by flow cytometry was then performed. Basing on the cellular cytotoxicity assay results, effector:target (5.0:1) showed the powerful cytotoxicity of NK cells, So the coculture cell ratio will be employed in the further FACS array. The results indicated that, compared with those in the control group, the numbers of early and late apoptotic cells were significantly increased in the NK cell-treated groups. The apoptosis-inducing effects of NK cells on H460 cells (9.23 ± 2.32% vs. 2.6 ± 0.614%, *p* < 0.05, Fig. 1B) and Calu-1 cells (21.9 ± 3.6% vs. 2.9 ± 0.85%, *p* < 0.05, Fig. 1C) were significant. Similar to the cellular cytotoxicity assay results, H460 cells showed a lower apoptosis rate than Calu-1 cells (Fig. 1D). These results showed that different NSCLC cell lines showed distinctive susceptibility to NK cell-mediated lysis.

### NK cells lyse NSCLC cells in a Granzyme B-dependent manner

**Fig. 2:**
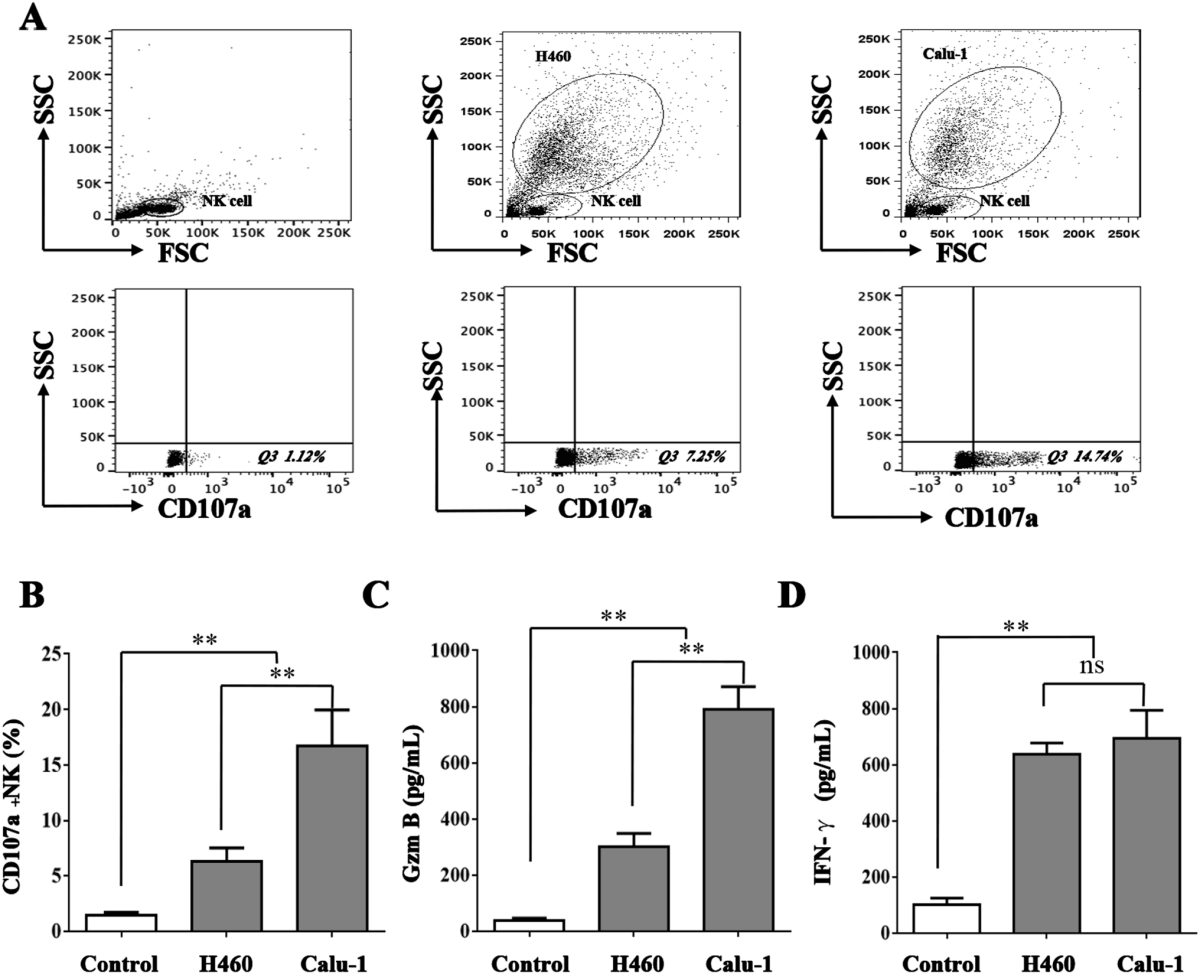
Detection of cytotoxicity of NK cells on NSCLC cells. NK cells were stained for surface expression of CD107a and intracellular expression of Granzyme B and IFN-γ after coculture with H460 and Calu-1 cells. CD107a^+^ NK cells percentage was determined by FACS **A** and **B**. Granzyme B **C** and IFN-γ **D** concentrations in the cell culture supernatant were measured by Elisa. The data are expressed as the mean ± the standard deviation of three experiments. ns *p*0.05, *p < 0.05 and **p < 0.01 vs. control

**Fig. 3:**
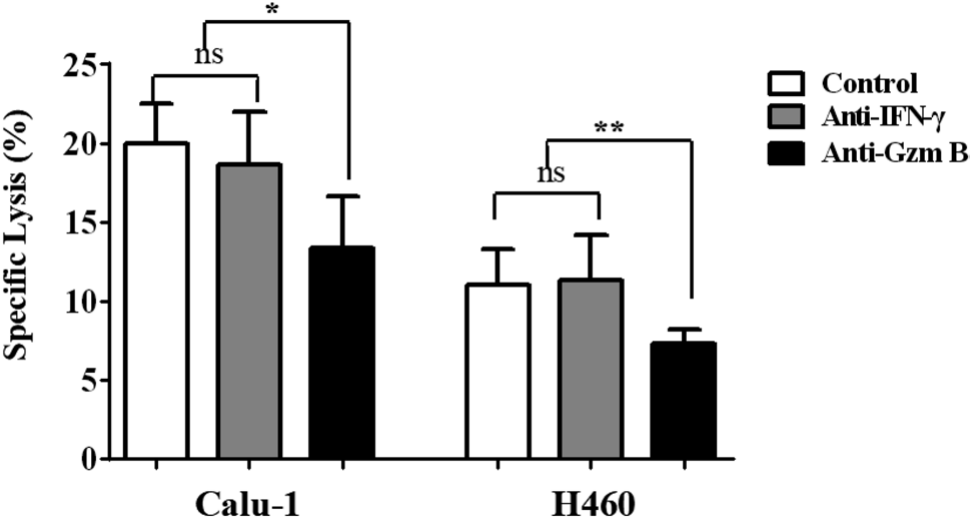
NK cells lyse NSCLC cells in a Granzyme B-dependent manner. Granzyme B and IFN-γ antibody were added to the NK cells/NSCLC cells coculture systems. H460 and Calu-1 cells were treated with NK cell on E:T = 5:1. NK cell-mediated cellular cytotoxicity was determined using a non-radioactive cellular cytotoxicity assay kit. ns *p*0.05, *p < 0.05 and **p < 0.01 vs. control

When NK cells were cocultured with NSCLC cells, CD107a, a cytotoxicity marker on NK cells, was markedly expressed. As shown in Fig. 2A and B, CD107a^+^ NK cells accounted for 16.2 ± 2.5% (*p* < 0.05) and 8.3 ± 1.4% (*p* < 0.05) of the NK cells in the cocultures with Calu-1 and H460 cells, respectively. Granzyme B and IFN-γ concentrations in the cell culture supernatant were measured by ELISA. As shown in Fig. 2C and D, consistent with CD107a expression, Granzyme B and IFN-γ secretion was elevated in coculture with NSCLC cells. Unlike Granzyme B and CD107a expression, IFN-γ secretion in the two NSCLC cell lines was comparable (*p* > 0.05). Granzyme B may be the key factor in the cytolytic activity of NK cells toward NSCLC cells. To prove this hypothesis, anti-Granzyme B and anti-IFN-γ antibodies were added to the NK cell/NSCLC cell coculture system. As shown in Fig. 3, the cytolytic activity of NK cells toward Calu-1 and H460 cells was moderated when Granzyme B was blocked (*p* < 0.05). In contrast, regardless of whether the anti-IFN-γ antibody was added, the cytolytic activity did not differ (*p* > 0.05). The results showed that NK cells kill NSCLC cells in a Granzyme B-dependent manner.

### The expression of PD-L1 on H460 cells is higher than that on Calu-1 cells

**Fig. 4:**
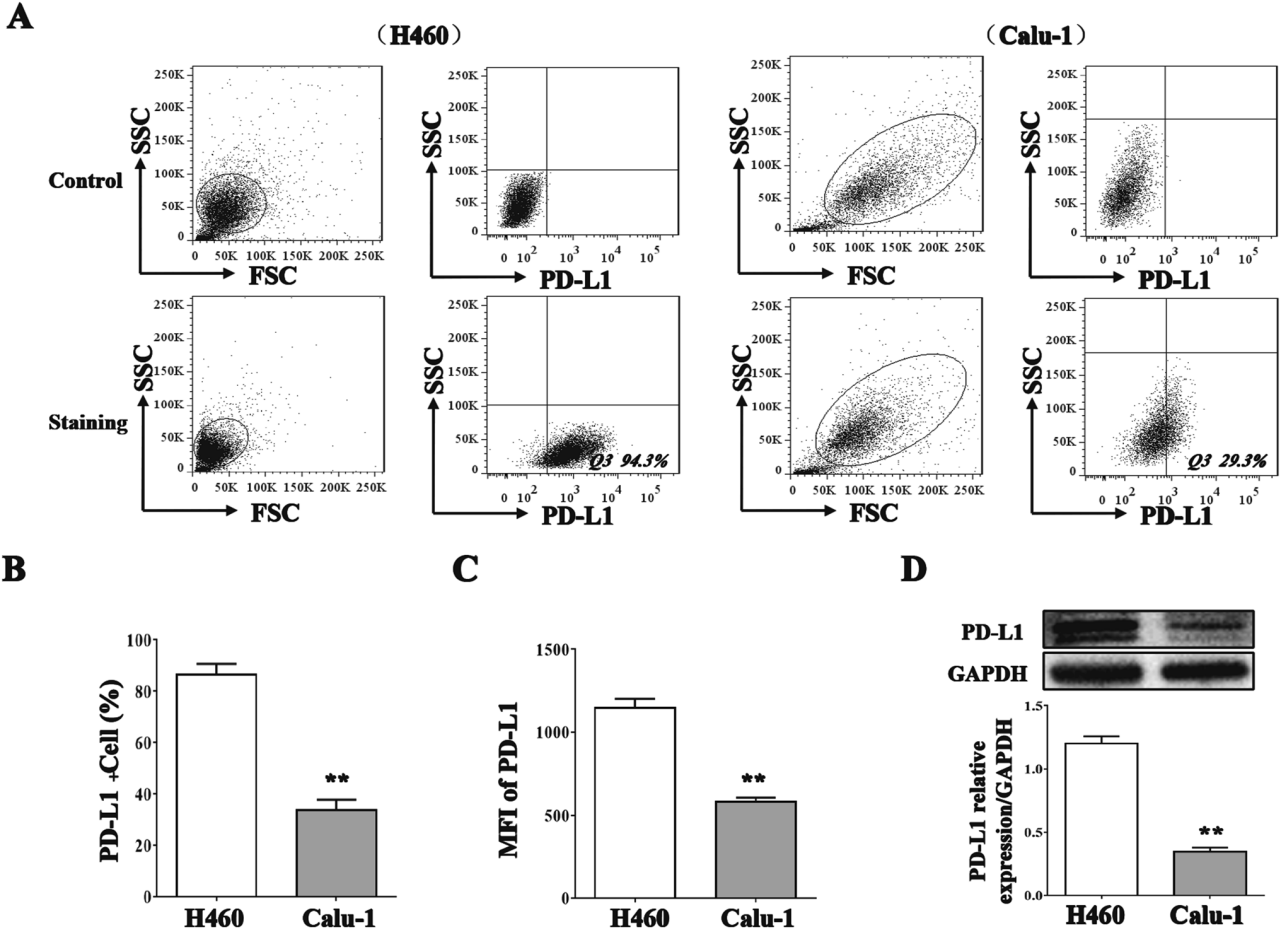
The expression of PD-L1 on H460 and Calu-1 cells. The PD-L1 positive cell percentage and the Mean fluorescence intensity (MFI) of PD-L1 on H460 and Calu-1 cells was quantified by FACS **A**–**C**. The protein expression on H460 and Calu-1 cells was quantified by western blotting **D**. The data are expressed as the mean ± the standard deviation of three experiments. **p < 0.01 vs. control

Tumor cells express PD-L1 to inhibit the cytolytic activity of NK cells because NK cells express PD-1. The expression of PD-L1 on H460 and Calu-1 cells was investigated by FACS and western blotting. The PD-L1-positive cell percentage and the mean fluorescence intensity (MFI) of PD-L1 on H460 cells were greater than those for Calu-1 cells (Fig. 4A–C, *p* < 0.01). The western blot results revealed that PD-L1 protein expression on H460 cells was threefold higher than that on Calu-1 cells (Fig. 4D, *p* < 0.01).

### PD-L1 blockage enhanced the cytotoxicity of NK cells on H460 cells

**Fig. 5:**
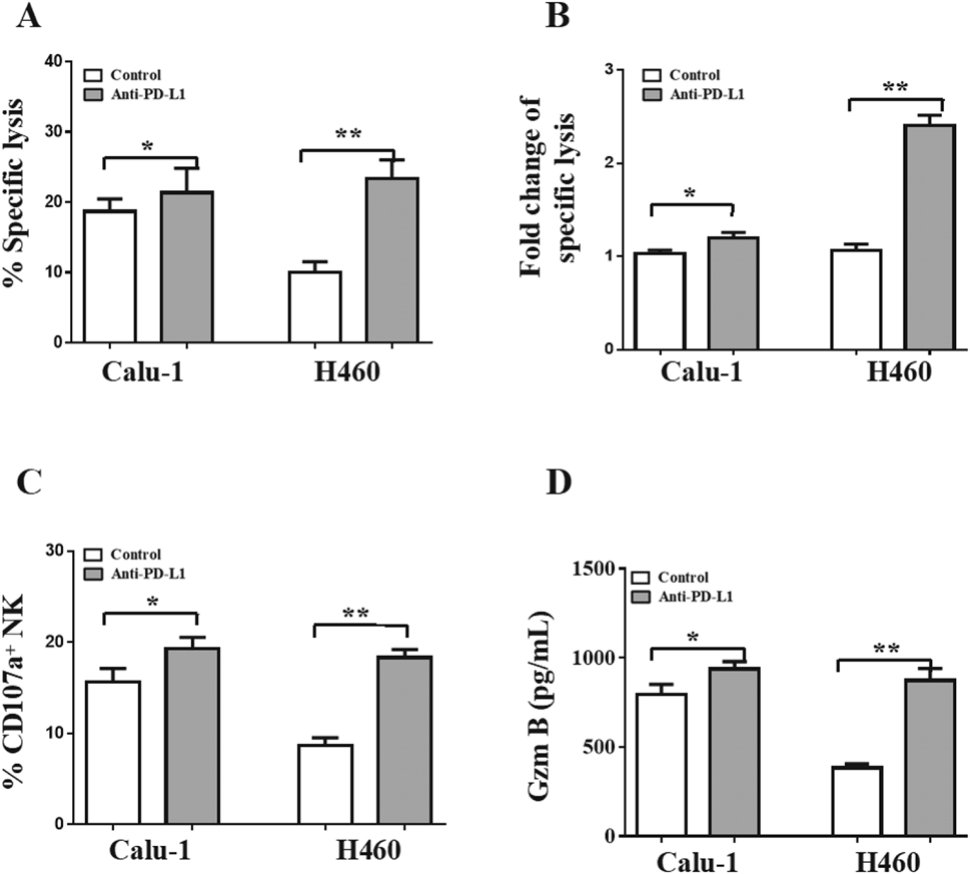
PD-L1 blockage enhanced the cytotoxicity of NK cells on H460 cells. PD-L1 antibody were added to the NK cell/NSCLC cell coculture systems. NK cell-mediated cellular cytotoxicity on H460 and Calu-1 cells **A**. The fold change of specific lysis on NSCLC cells when PD-L1 antibody added or not **B**. CD107a^+^ NK cells percentage **C**. Granzyme B concentrations in the cell culture supernatant **D**. The data are expressed as the mean ± the standard deviation of three experiments. *p < 0.05 and **p < 0.01 vs. control

To investigate the pivotal role of the PD-L1/PD-1 checkpoint in the cytotoxicity of NK cells toward NSCLC cells, an anti-PD-L1 antibody was added to the NK cell/NSCLC cell coculture system. The specific lysis of NSCLC cells by NK cells was enhanced when the PD-L1/PD-1 interaction was blocked with the anti-PD-L1 antibody (Fig. 5A, *p* < 0.05). Interestingly, the fold change in the specific lysis of H460 cells was much higher than that in the specific lysis of Calu-1 cells due to the higher expression of PD-L1 on H460 cells than on Calu-1 cells (Fig. 5B, *p* < 0.01). The CD107a expression (Fig. 5C, *p* < 0.05) and Granzyme B secretion (Fig. 5D, *p* < 0.05) levels of NK cells were also increased in the coculture systems with each cell line.

### NK cells from NSCLC patients express high levels of PD-1

**Fig. 6:**
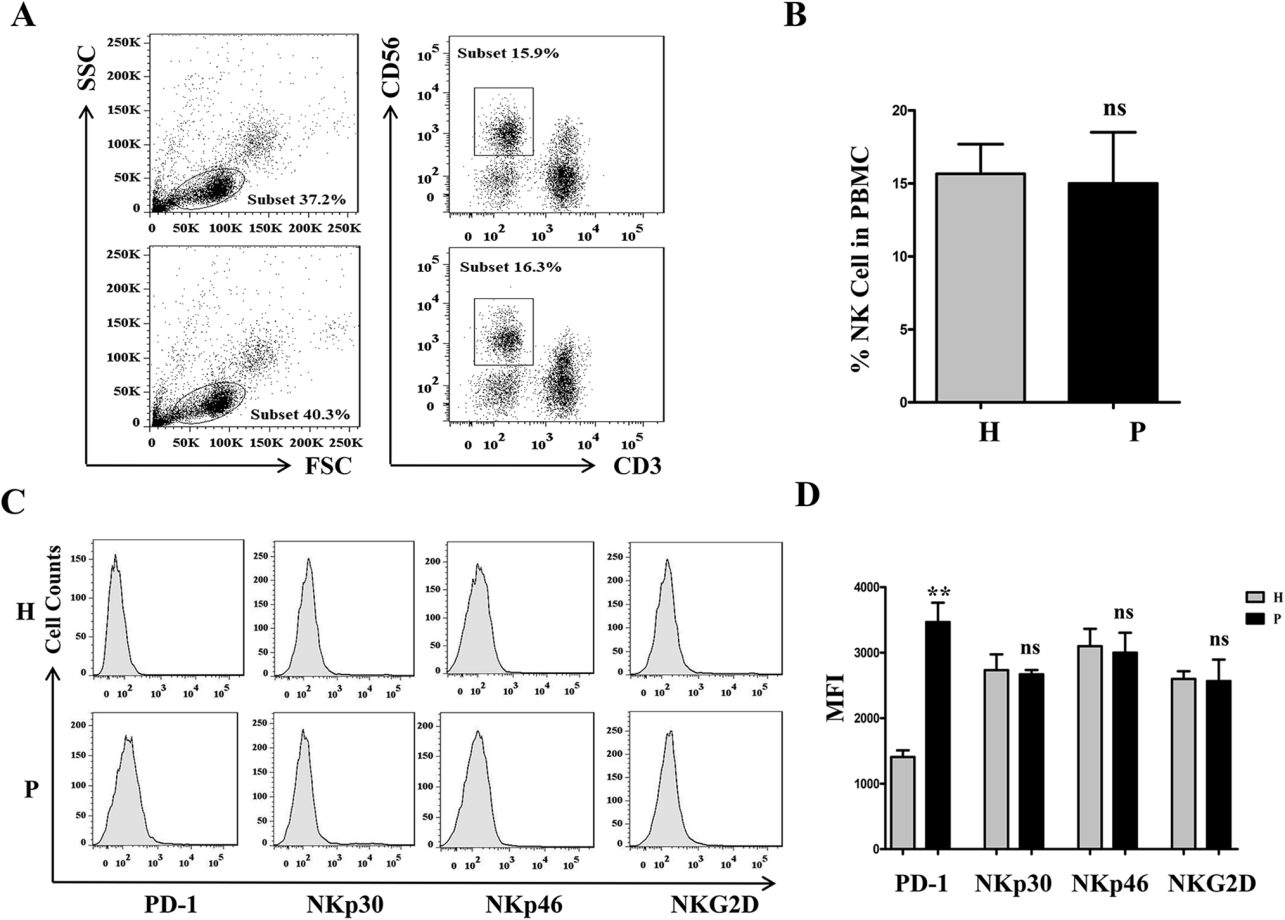
NK cells from NSCLC patients express high level of PD-1. Freshly isolated PBMCs from 10 healthy donors and 10 NSCLC patients were stained for detection of NK cell percentage and function. NK cell percentages in PBMC stained by CD3 and CD56 antibody **A** and **B**. The expression levels of PD-1, NKp30, NKp46 and NKG2D on NK cells **C** and **D**. P is abbreviation of patients, H is abbreviation of healthy donor. The data are expressed as the mean ± the standard deviation of three experiments. *ns *p*0.05 and **p < 0.01 vs. control

Freshly isolated PBMCs from 10 healthy donors and 10 NSCLC patients were stained for assessment of the NK cell percentage and NK cell function. The percentage of NK cells among the PBMCs was approximately 15% in healthy donors and NSCLC patients, and there were no differences, as shown in Fig. 6A and B (*p* > 0.05). NK cell surface receptor detection revealed that the expression levels of PD-1 on NK cells from NSCLC patients were significantly higher than those on NK cells from healthy controls (Fig. 6C and D, *p* < 0.01). However, the expression levels of NKp30, NKp46 and NKG2D on NK cells were similar in the patients and the healthy donors (Fig. 6C and D, *p* > 0.05).

### PD-L1 blockage enhances the cytotoxicity of NK cells isolated from NSCLC patients in H460 cells

**Fig. 7:**
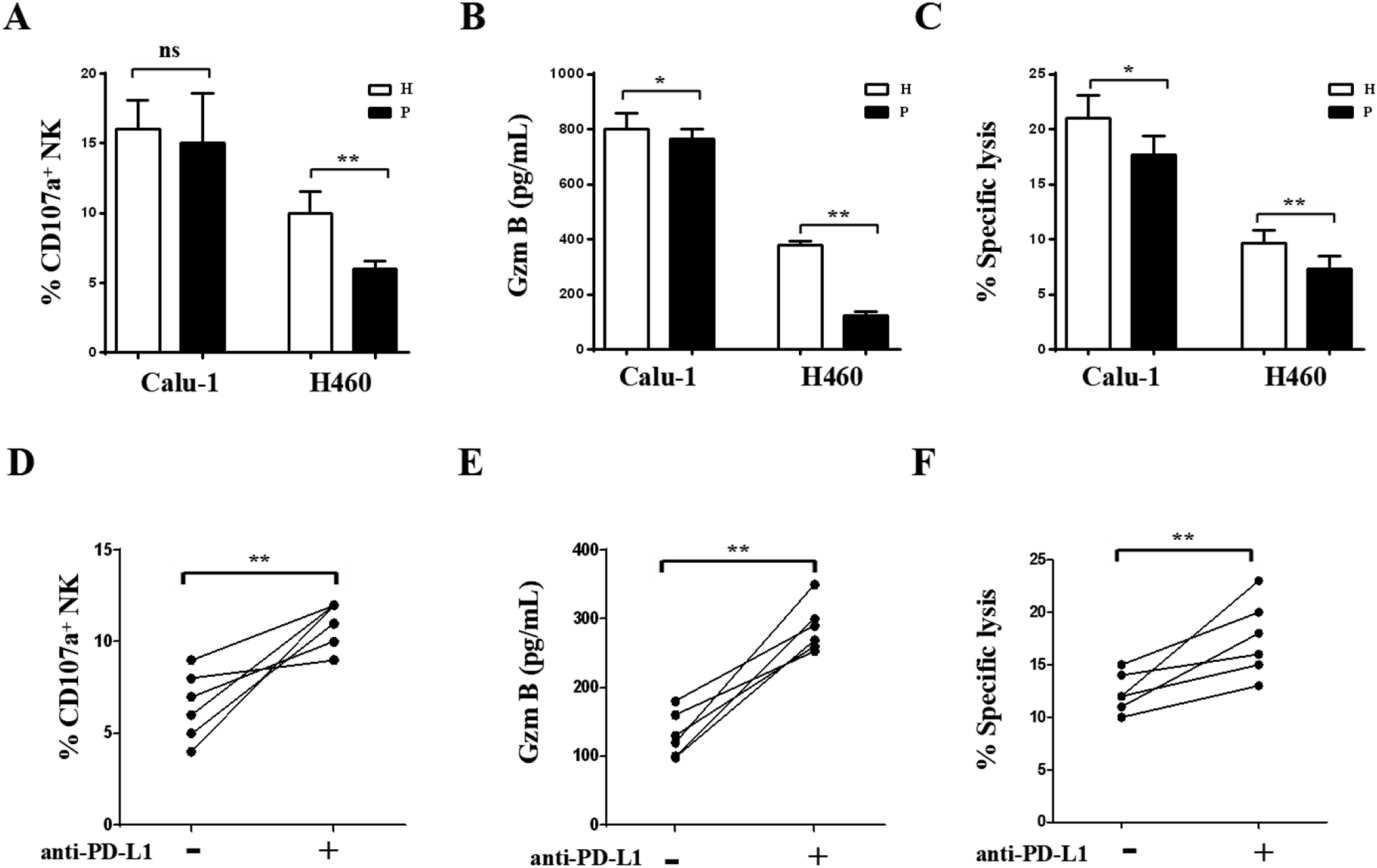
PD-L1 blockage enhance the cytotoxicity of NK cells from NSCLC patients to H460 cell lines. NK cells were purified from 5 NSCLC patients or 5 healthy donors and cultured with H460 or Calu-1 cell lines for cytotoxicity detection. CD107a^+^ NK cell percentages **A**, Granzyme B concentrations **B**, and the lysis of H460 or Calu-1 cells **C** were detected. NK cells from 5 NSCLC patients cultured with H460 cells with or without PD-L1 antibody for function detection. CD107a^+^ NK cell percentages **D**, the Granzyme B secretion **E**, the lysiss of H460 cells **F** were detected when PD-L1/PD-1 blockage by PD-L1 antibody. *P* patients, *H* is healthy donor. The data are expressed as the mean ± the standard deviation of three experiments. ns *p*0.05, *p < 0.05 and **p < 0.01 vs. control

**Fig. 8:**
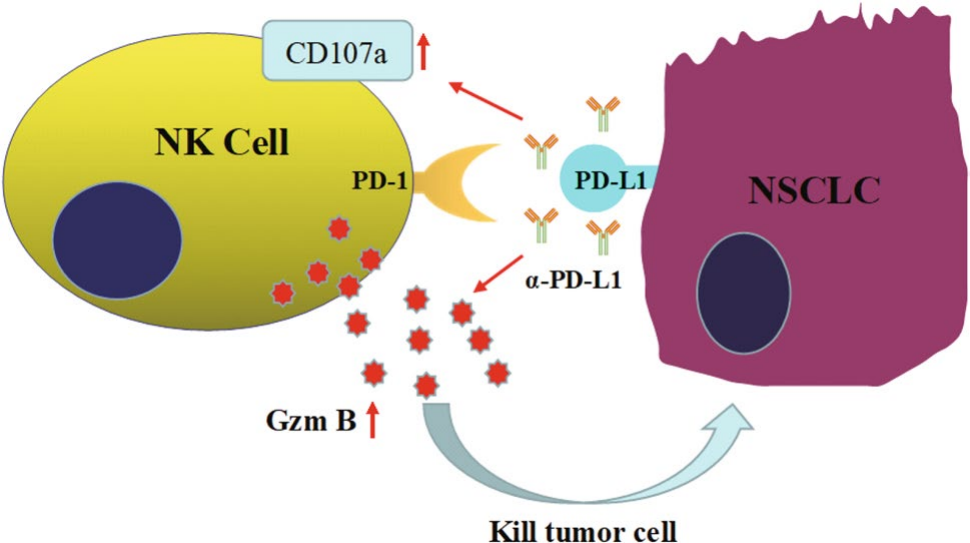
Schematic outline of PD-L1/PD-1 blockage enhanced the cytotoxicity of natural killer cells on the NSCLC cells by Granzyme B secretion. NK cells kill the NSCLC cells which are lack of MHC molecules by exerting cytolytic functions mainly through secreting Granzyme B and expressing CD107a. But some NSCLC cell lines, or lung cancer patients, over expression of PD-L1 molecular, interacting with PD-1 on NK cells to escape from killing. When PD-L1/PD-1 was blocked by PD-L1 antibody, NK cells restore the killing ability against lung cancer by means of expressing CD107 and Granzyme B secretion

NK cells isolated from 5 NSCLC patients or 5 healthy donors were purified and cultured with H460 and Calu-1 cells for cytotoxicity analysis. The percentages of CD107a^+^ NK cell percentages from NSCLC patients and healthy donors were similar in coculture with Calu-1 cells. Interestingly, in coculture with H460 cells, the percentages of CD107a^+^ NK cells from NSCLC patients were significantly lower than those from healthy controls (Fig. 7A, *p* < 0.01). Granzyme B concentrations in the cell culture supernatant were measured by ELISA. As shown in Fig. 7B, consistent with CD107a expression, Granzyme B secretion by the NK cells isolated from patients with NSCLC was decreased compared with that by the NK cells isolated from healthy controls in coculture with H460 cells (*p* < 0.01). Lysis of H460 cells was decreased when the cells were cocultured with NK cells from patients compared with those from healthy controls (Fig. 7C, *p* < 0.01). This phenomenon is due to the PD-L1/PD-1 interaction between NK cells and H460 cells. NK cells from 5 NSCLC patients were cultured with H460 cells in the presence or absence of an anti-PD-L1 antibody for functional assessment. As expected, the CD107a^+^ NK cell percentage (Fig. 7D, *p* < 0.01), Granzyme B secretion (Fig. 7E, *p* < 0.01), and apoptosis of H460 cells (Fig. 7F, *p* < 0.01) were increased when the PD-L1/PD-1 interaction was blocked with the anti-PD-L1 antibody. These results showed that PD-L1 expression on NSCLC cells plays an important role in the susceptibility to NK cell-mediated lysis. The PD-L1/PD-1 interaction plays an important role in tumor escape from immunosurveillance (Fig. 8).

## Discussion

The relationship among the host immune system, the kind of cancer and its treatment is extremely complex [27, 28]. The recent success of immunotherapy in the laboratory and in other malignant tumors has highlighted the potential of immune-based therapeutic approaches for NSCLC [29, 30]. As a main component of the innate immune system, NK cells can kill tumor cells or infected cells directly and are not dependent on antigen-presenting cells. CD56^dim^CD16^bright^ NK cells (approximately 90%) and CD56^bright^CD16^dim^ NK cells are two subsets of NK cells in the peripheral blood of healthy adults. CD56^dim^CD16^bright^ NK cells exert primarily cytolytic functions mainly through secretion of granzyme B and perforin, whereas CD56^bright^CD16^dim^ NK cells exert primarily immunoregulatory functions by secreting cytokines [31]. Cells lacking MHC molecules can activate NK cells by interacting with activating receptors on the cell surface, including NKp30, NKp40, NKG2D and NKp46. Tumor cells are more susceptible to NK cell-mediated lysis due to their lack of MHC class I molecules [32]. The exocytosis of lytic granules is a multistep regulated process that is initiated by the contact between the effector and the target cell [33], leading to the formation of an immunological synapse [34], lytic granules fuse with the plasma membrane and release their content into the immunological synapse. Lytic granules contain the pore-forming protein perforin and several proteases called granzymes, of which granzyme B is the best characterized one [35]. The expression of CD107a is used to evaluate the degranulation ability of NK cells [36]. In this study, we tested two NSCLC cell lines, Calu-1 and H460, for susceptibility to the cytolytic activity of freshly isolated NK cells from healthy donors. Both cell lines were lysed by NK cells in a dose-dependent manner. H460 cells showed less susceptibility to NK cell-mediated lysis than Calu-1 cells at all ratios. This finding indicated that NSCLC cells, similar to other malignant cells, are more susceptible than nonmalignant cells to NK cell-mediated lysis. However, the expression of specific inhibitory molecules differs among cell lines and patients.

PD-L1, an immune checkpoint molecule, plays an important role in tumor escape from immunosurveillance. PD-1, a receptor for PD-L1, is expressed on immune cells, including NK cells, and interacts with tumor cells, leading to apoptosis, anergy or tolerance. PD-L1 expression appears to be conserved across a number of solid tumors and hematologic malignancies. The PD-L1 protein is expressed in a variety of cancers, such as melanoma, non-small cell lung cancer, and lymphoma [37]. PD-L1 protein expression on the tumor cells of patients, as detected by IHC, is a predictor of the response to both anti-PD-L1 and anti-PD-1 therapy in a variety of cancers [23, 24]. To prove the reason for the difference in cytolytic activity towards H460 cells and Calu-1 cells, the expression of PD-L1 on H460 cells and Calu-1 cells was investigated. PD-L1 expression was quantified by FACS and western blotting. PD-L1 protein expression on H460 cells was greater than that on Calu-1 cells. To investigate the pivotal role of the PD-L1/PD-1 checkpoint on the cytotoxicity of NK cells in NSCLC cells, an anti-PD-L1 antibody was added to the NK cell/NSCLC cell coculture system. The specific lysis NSCLC cells by NK cells was enhanced when the PD-L1/PD-1 interaction was blocked with the anti-PD-L1 antibody.

To further address the specific mechanism by which NK cells lyse NSCLC cells, CD107a expression, Granzyme B secretion and IFN-γ secretion were evaluated. When NK cells were cocultured with NSCLC cells, CD107a was markedly expressed. Granzyme B and IFN-γ concentrations in cell culture supernatants were also measured by ELISA. Unlike Granzyme B secretion and CD107a expression, IFN-γ secretion was comparable in both NSCLC cell lines. Granzyme B may be the key factor in the cytolytic activity of NK cells toward NSCLC cells. To prove this hypothesis, anti-Granzyme B and anti-IFN-γ antibodies were added to the NK cell/NSCLC cell coculture system. The cytolytic activity of NK cells toward NSCLC cells was moderated when Granzyme B was blocked. In contrast, regardless of whether the anti-IFN-γ antibody was added, the cytolytic activity of NK cells toward NSCLC cells did not differ. These results showed that NK cells lyse NSCLC cells in a Granzyme B-dependent manner. We also determined the NK cell percentage and assessed the expression of cell surface receptors in NSCLC patients and healthy donors. There were no differences in the NK cell percentages between healthy donors and NSCLC patients, but the expression levels of PD-1 on NK cells from NSCLC patients were significantly higher than those on NK cells from healthy controls. This phenotype resulted in lower cytolytic activity toward the H460 cell line, which has high PD-L1 expression, but not toward Calu-1 cells. This phenomenon was proven by a PD-L1/PD-1 blockade experiment.

For patients who are not candidates for surgery, immunotherapy is a promising therapeutic option for advanced NSCLC [38]. Expansion of autologous tumor-specific effector cells ex vivo before infusion into the host plays an important role in adoptive cell immunotherapy [39]. The immunological characteristics of cancer cells are also an important indicator for therapy selection. In summary, the present study revealed that PD-L1/PD-1 blockade enhanced the cytotoxicity of natural killer cells in NSCLC via Granzyme B secretion. Whether anti-PD-L1 and anti-PD-1 therapy will be used depends on PD-L1 protein expression in the tumors of patients as assessed by IHC. This study will facilitate the precise treatment of lung cancer.

## Data Availability

The data generated or analysed during this study are included in this article.
